# Effects of early energy intake on neonatal cerebral growth of preterm newborn: an observational study

**DOI:** 10.1038/s41598-021-98088-4

**Published:** 2021-09-16

**Authors:** Giovanni Boscarino, Maria Di Chiara, Raffaella Cellitti, Maria Chiara De Nardo, Maria Giulia Conti, Pasquale Parisi, Alberto Spalice, Chiara Di Mario, Benedetta Ronchi, Alessia Russo, Francesca De Luca, Ida Pangallo, Gianluca Terrin

**Affiliations:** 1 Department of Maternal and Child Health, Sapienza University of Rome-Policlinico Umberto I, Viale del Policlinico 155, 00161 Rome, Italy; 2 Department of Molecular Medicine, Sapienza University of Rome, Rome, Italy; 3 Chair of Pediatrics, NESMOS Department, Faculty of Medicine and Psychology, Sapienza University of Rome, Rome, Italy

**Keywords:** Neuroscience, Gastroenterology, Neurology

## Abstract

Current guidelines for preterm newborns recommend high energy nutrition soon after birth in order to limit growth retardation. However, long-term effects of this nutritional approach are still debated, and it has been demonstrated that cerebral growth depends on protein intake in early life. A negative impact of early high energy intake by parenteral nutrition (PN) has been reported for patients in critically ill conditions, observed in intensive care unit. We aimed at evaluating the impact of energy intake on cerebral growth in preterm neonates early in life. We included preterm newborns with gestational age < 32 weeks or birth weight (BW) < 1500 g. Measurement of cerebral structures was performed by cranial Ultrasonography (cUS) between 3 and 7 days of life (DOL, T0) and at 28 DOL (T1). We evaluated the relation between energy intake and cerebral growth in the first 28 DOL. We observed in 109 preterm newborns a significant (*p* < 0.05) negative correlation between energy intake received by PN and right caudate head growth (*r* = − 0.243*) and a positive correlation between total energy intake and transverse cerebellum diameter (*r* = 0.254*). Multivariate analysis showed that energy intake administered by enteral nutrition (EN), independently increased growth of left caudate head (β = 0.227*) and height cerebellar vermis (β = 0.415*), while PN independently affected growth of both right and left caudate head (β = − 0.164* and β = − 0.228*, respectively) and cerebellum transverse diameter (β = − 0.849*). The route of energy administration may exert different effects on cerebral growth in early life. High energy intake administered through EN seems to be positively correlated to cerebral growth; conversely, PN energy intake results in a poorer cerebral growth evaluated with cUS.

## Introduction

Preterm newborns are exposed to a high risk of faltering growth, mainly due to undernutrition. It has been demonstrated that growth restriction is associated with adverse neurodevelopment (NDV) outcomes^1^. Thus, preterm newborns have an increased risk of less than optimal NDV compared with their term-born counterparts^2^.

In order to limit growth retardation, current guidelines for preterm neonates recommend the administration of high doses of protein and energy intakes through parenteral nutrition (PN), starting soon after birth^3,4^. Even though optimizing early nutritional intake in preterm neonates may reduce growth restriction, its effects on brain development, in early life, are scarce and still largely controversial^5^.

Infants born during late second and third trimesters of pregnancy are at a time of critical brain development and have evidence of impaired brain maturation, as reflected in altered brain size^6^. Cerebral growth in preterm infants might be influenced by different nutritional strategies administered early in life^7–9^. There is emerging evidence that early protein intake has a positive impact on cerebral size^10^, as demonstrated by magnetic resonance imaging (MRI). Increasing evidence indicates that cranial ultrasound (cUS) is a valid methodic to study neonatal brain and easier to be performed in preterm infants compared to MRI^11–14^. In a observational study, we have recently demonstrated that the route of protein administration has a significant impact on cerebral size on cUs during neonatal life^8^. In particular, we observed that high protein supply negatively affects cerebral measurements when administered by PN. On the other hand, protein intake given by enteral nutrition (EN) seems to be associated with a greater size of cerebral structures, such as cerebellum and caudate, at 28 days of life (DOL)^8^. Previous studies have mainly focused on the effects of protein intake, whilst the impact of the sole early energy intake on neonatal brain has yet to be investigated. In light of these considerations, we aimed to evaluate the influence of energy intake, received in the first DOL by EN or PN, on brain growth, in preterm neonates.

## Methods

### Study design and population

We designed a prospective observational study to assess the effects of energy intake on brain measurements by using 2-D cUS in preterm neonates. All preterm newborns with gestational age (GA) < 32 weeks or body birth weight (BW) < 1500 g, consecutively admitted to the Neonatal Intensive Care Unit (NICU) of Policlinico Umberto I Hospital, Sapienza University of Rome, were prospectively included between May 2017 and May 2020. We excluded infants with major congenital intestinal and extraintestinal diseases, inborn errors of metabolism, family history of allergy, use of pre-or probiotics, congenital infections, intraventricular haemorrhage (IVH), periventricular leukomalacia (PLV), death or transfer to other hospital before 72 h of life^15–21^. Of note, the majority of the included neonates were also included in a previous study^8^ with additional neonates born between September 2019 and May 2020. The study was conducted in conformity with World Medical Association Declaration of Helsinki for medical research involving human subjects, and it was approved by Ethics Committee of Policlinico Umberto I, Sapienza University of Rome (with number 5089). Informed written consent was obtained from all parents.

### Collection data

We prospectively collected prenatal, perinatal, and postnatal information for each patient in a specific data form. In particular, GA, BW, gender, type of delivery, twin pregnancy, antenatal steroid administration, Apgar score at 5 min after birth, pH on cord blood at birth, body temperature in the 1st hour after birth, death and need of invasive mechanical ventilation were recorded. We performed, according to standard criteria, diagnosis of the major morbidities associated with prematurity including necrotizing enterocolitis (NEC, Bell stage ≥ 2), bronchopulmonary dysplasia (BPD, moderate grade), retinopathy of prematurity (ROP, stage ≥ 2) and sepsis proven by positive cultures; we reported the diagnosis in a pre-specified data form, as previously described^22–26^. Data on daily enteral and parenteral nutritional intake, were collected, during the first week of life.

### Nutritional protocol

Administration of nutrient supply was performed following the nutritional protocol, as previously described (Supplementary Table 1)^8^. Human milk (HM) of own mother or preterm formula (PF) was administered as soon as possible after birth. The PF was administered to the infants when HM was not available or sufficient. Minimal enteral feeding was commenced at 10–20 ml/kg/day. The amount was increased by 20–30 ml/kg/day if EN was tolerated^27,28^. In case of feeding intolerance, EN was suspended^18^. No changes were made regarding enteral feeding policy during the two study periods.

The PN, started early after birth, was administered via central vascular access to guarantee an adequate intake of fluids, electrolytes and nutrient until full enteral feeding (120 kcal/Kg/day) was achieved^29^.

The overall fluid intake (EN + PN) was started with 70–90 ml/kg/day and was increased by 10–20 ml/kg/day until the achievement of 150–180 ml/ kg/day, which was aimed to be reached by 7 to 10 DOL^30^. Preterm HM was assumed to contain 65 kcal/100 ml (1.5 g of protein/100 ml, 3.5 g of fat/100 ml, 6.9 g of carbohydrate/100 ml)^31^. Macronutrients’ content of formula (Pre-Nidina Nestlè, Milan, Italy) and of PN were calculated based on the published manufacturer’s labels, including proteins (TrophAmine 6% Braun Medical Inc. Irvine, USA), lipids (Smoflipd, Fresenius Kabi, USA), and carbohydrates (Dextrose injection 10%, Fresenius Kabi, USA) expressed in g/kg/day^32^.

### Cranial ultrasonography examination

We performed all the cUS measurements with an automatic multifrequency transducer (Philips Affiniti 50G, Andover, MA, USA) set between 5 and 10 MHz^33^. Anterior fontanel was the preferred acoustic window for the majority of measurements, in coronal and sagittal planes, according to standard procedures. Cerebellum and cerebellar vermis were evaluated using the mastoid fontanel on the axial plane. The cUS scans were performed in the first 24 h of life, between 3 and 7 DOL (according with compliance of the babies), at 14 and 28 DOL, by two examiners with high expertise (more than 10 years) in cUS, unaware of the nutrition protocol and study aims (R.C. and M.C.D.N.). Cerebral measurements were collected during the examinations performed at 3–7 DOL (T0) and at 28 DOL (T1). Cerebral structures were measured as previously described, with the infant’s head in supine position following the standard protocol^8^. With the anterior fontanel used as an acoustic window, standard views were obtained in the coronal and sagittal planes. Maximum length of corpus callosum (Fig S1) was measured in the midsagittal plane tracing a horizontal line between the extreme margins of the genu and the splenium. Maximum width of corpus callosum was measured in the midsagittal plane, separately for genu, body, and splenium. We visualized caudate nucleus below the floor of the frontal horn of the lateral ventricle, as a hypoechoic area located anteriorly to the caudothalamic groove. Width of the caudate head was measured in the parasagittal plane as the maximum extension of this area (Fig S2). Both height and width of the cerebellar vermis and transverse cerebellar diameter were measured in axial plane (Fig S3).

### Statistical analysis

Data analysis was performed using IBM the Statistical Package for the Social Sciences Statistics version 25.0 (SPSS Inc-IBM Corp, Chicago, IL). We checked for normality using Shapiro–Wilk test. The mean and standard deviation summarized continuous variables. We compared categorical variable using χ^2^ test and paired and unpaired variables by t-test or Mann–Whitney. We calculated the cerebral growth from T0 to T1 [(T1 − T0)/T0]. Nutritional intake was related to the growth measurements of the different brain structures of the first 28 DOL. We performed correlation between variables by Wilcoxon rank sum tests and by Pearson correlation.

Multivariate regression analysis was performed to study the possible influence of confounding variables (i.e., BW, gender, pH on cord blood, morbidity and energy intake thought EN or PN) on linear measurements of cerebral structures at 28 DOL. The level of significance for all statistical tests was 2-sided (*p* < 0.05).

### Ethical approval

The study was conducted in conformity with World Medical Association Declaration of Helsinki for medical research involving human subjects, and it was approved by Ethics Committee of Policlinico Umberto I, Sapienza University of Rome (with number 5089).

### Consent to participate

Informed consent was obtained from all parents of newborn.

## Results

**Figure 1:**
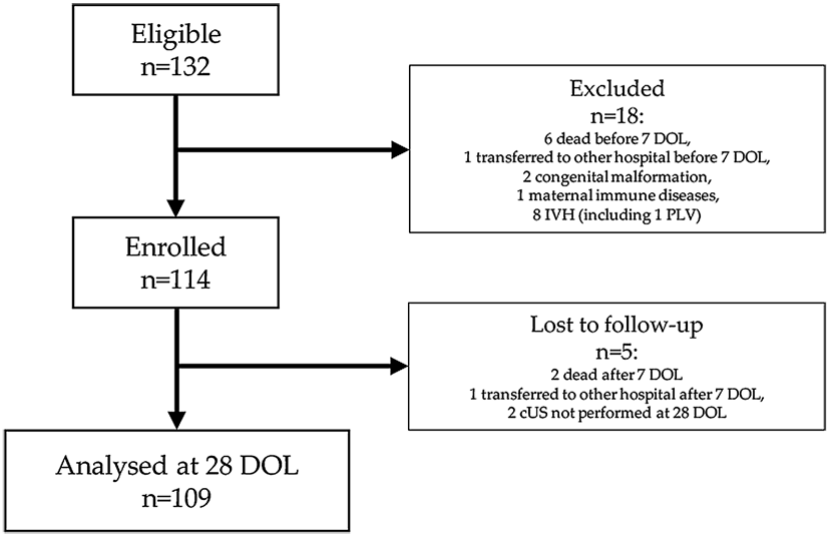
Flow-chart. *DOL* days of life, *IVH* intraventricular hemorrhage, *PLV* Periventricular Leukomalacia, *cUS* cranial ultrasound.

We enrolled 114 preterm newborns and we analysed 109 preterm newborns, as showed in Fig. 1. In the Table 1 we showed the main clinical characteristics of the study population. The cerebral size at birth were showed in Supplementary Table 2.

**Table 1:** Clinical characteristics of study population.

N. 109	
Gestational age, *weeks*	29 ± 2
Birth weight, g	1288 ± 362
Female, *N. *(%)	50 (45.9)
Cesarean section, *N. *(%)	97 (89.0)
Twins, *N. *(%)	36 (33.0)
Antenatal steroids^a^, *N. *(%)	85 (78.0)
5-min Apgar score	8 ± 1
pH at birth	7.3 ± 0.1
Temperature at the 1st hour, *°*C	36.2 ± 0.5
Mortality, *N. *(%)	2 (1.8)
Invasive mechanical ventilation, *N. *(%)	27 (24.8)
NEC Bell stage ≥ 2, *N. *(%)	4 (3.7)
BPD moderate grade, *N. *(%)	5 (4.6)
ROP stage ≥ 2, *N. *(%)	8 (7.3)
Sepsis proven by positive cultures, *N. *(%)	9 (8.3)
Full enteral feeding, *days of life*	14 ± 13
Star of enteral nutrition, *days of life*	1 ± 1
Duration of parenteral nutrition, *days*	14 ± 13

*NEC* necrotizing enterocolitis bell stage ≥ 2, *BPD* bronchopulmonary dysplasia, *ROP* retinopathy of prematurity.

Data were expressed as mean ± standard deviation, when not specified.

^a^Intramuscular steroids cycle in two doses of 12 mg over a 24-h period.

We observed a correlation between cerebral growth and energy intake of the first week of life (Table 2). In particular, we observed a negative correlation between energy intakes received by PN and right caudate head and between total energy intakes and transverse diameter of cerebellum (Table 2).

**Table 2:** Correlations between cerebral structures linear measures and energy intake.

Growth measures	Energy Intake in the 1st week of life		
	by EN (kcal/Kg/firstWeek)	by PN (kcal/Kg/firstWeek)	Total (kcal/Kg/firstWeek)
**Corpus Callosum**			
Length	0.048	0.135	0.218
Body	0.022	− 0.118	− 0.138
Genu	0.210	− 0.129	0.006
Splenium	0.073	− 0.059	− 0.016
**Caudate head**			
Right	0.117	− 0.243*	− 0.223
Left	0.206	− 0.074	0.074
**Cerebellum**			
Transverse diameter	0.193	0.070	0.254*
Vermis height	0.098	0.058	0.159
Vermis width	0.105	− 0.068	− 0.002

(T1 − T0)/T0.

*EN* enteral nutrition, *PN* parenteral nutrition.

*p < 0.05.

Multivariate analysis showed that energy intake in the first week of life, given by EN, was an independent risk factor influencing, positively, the cerebral growth of left caudate head width and height of cerebellar vermis (Table 3). Regression analysis also showed that energy intake administered by PN, independently and negatively affected growth of caudate head width (right and left) and cerebellum transverse diameter (Table 3). The growth of length of corpus callosum was positively influenced by BW and energy intake by EN of the first week of life and negatively by the presence of at least one prematurity-related morbidity (Table 3).

**Table 3:** Multivariate analysis of covariate influencing cerebral growth measures of the first 28 days of life in preterm newborns.

Dependent variables°	Covariates (Model 1)					Covariates (Model 2)				
	Birth Weight	Gender	pH at birth	Morbidity^§^	EN Energy Intake 1st w	Birth Weight	Gender	pH at birth	Morbidity^§^	PN Energy intake 1st w
Corpus Callosum, Length	2.783*	− 0.707	− 2.867	− 3.193*	5.745*	1.329	− 0.401	− 3.565	− 3.057*	− 4.654
Corpus Callosum, Body	0.025	0.000	0.093	− 0.012	− 0.019	0.023	− 0.001	0.092	− 0.011	0.001
Corpus Callosum, Genu	0.043	0.008	0.042	0.009	0.079	0.075	0.010	0.056	− 0.001	0.040
Corpus Callosum, Splenium	0.024	− 0.010	− 0.047	0.011	0.072	0.079*	− 0.010	− 0.022	− 0.004	0.086
Caudate Head Width, Right	0.122*	0.016	0.074	0.003	0.038	0.045	0.021	0.038	0.020	− 0.164*
Caudate Head Width, Left	0.031	0.014	0.071	0.036	0.277*	− 0.041	0.029	0.037	0.043	− 0.228*
Cerebellum Transverse Diameter	0.691*	0.145	− 0.050	− 0.075	0.658	0.366	0.187	− 0.203	− 0.022	− 0.849*
Cerebellar Vermis, Height	0.069	0.049	− 0.240	0.028	0.415*	0.095	0.065	− 0.230	0.007	− 0.075
Cerebellar Vermis, Width	0.094	0.006	0.012	0.023	0.290	0.044	0.020	− 0.013	0.024	− 0.189

Growth measurement: (T1 − T0)/T0.

*EN* enteral nutrition, *PN* parenteral nutrition, *1st w* first week of life, ^§^*NEC* necrotizing enterocolitis and/or Sepsis proven by positive cultures and/or *BPD* bronchopulmonary dysplasia and/or *ROP* retinopathy of prematurity.

*p < 0.05.

## Discussion

We observed that cerebral growth of brain structures including corpus callosum, caudate head and cerebellum, may depend on early energy intake in preterm newborns. We demonstrated that the route of energy administration has different impact on cerebral growth. Specifically, administration of high energy intake through enteral route has positive effects on growth of transverse cerebellum diameter, left caudate head and height cerebellar vermis in neonatal age, whilst high amount of calories by PN adversely affects the early growth of both right and left caudate head and cerebellum transverse diameter.

Available evidence does not draw a definitive conclusion on the effects of early energy intake on brain growth. In brief, only few trials evaluated the effects of different nutritional strategies on cerebral growth in the first weeks of life. Tan et. al reported similar effects of two nutritional protocols, that slightly differed for energy (98.7 vs. 93.6 kcal/kg/day) and protein intake (2.6 vs. 2.3 g/kg/day), on brain size of preterm newborns with GA less than 29 weeks. Nevertheless, they did not compare the effects of energy intake received by EN versus PN. Similar results were reported by NEON trial^34^. In this study, authors did not find any effects of total energy intake on cerebral volumes. However, in this trial, the difference in energy intake was due mainly to the different amount of protein, given by PN, while, the role of enteral energy intake was not evaluated^34^. Besides, Isaacs et al. demonstrated a positive effect of higher energy intake on caudate nuclei, when measured in adult life, comparing two preterm formulas characterized by different protein (2 vs*.* 1.45 g/dl) and energy (80 vs. 68 kcal/dl) content given by EN^35^. Yet, the role of PN, in this trial was not investigated.

To the best of our knowledge, we separately evaluated, for the first time, the role of the route of administration of recommended energy intake on the brain development, in neonatal life of preterm newborns. Our results underline the importance of energy intake on cerebral size, but, at the same time, suggest caution in the administration of energy enhanced PN in the first DOL.

The tolerance of recommended energy intake given by PN in preterm newborns is still debated. Bonsante et al. demonstrated in an observational study that high PN energy intake is associated with metabolic acidosis^36^. Other studies have demonstrated an association between high energy intake in early life and hyperglycemia which is in turn associated with mortality and cerebral impairment of survived preterm newborns^37,38^.

Rising evidence supports the hypothesis that risks related to high energy intake may outweigh the benefits in critically ill subjects^39^. It has been recently described that high energy intake in PN, at early stages in critically ill patients, adversely affect neurological outcomes^39^. More recently, mitochondrial dysfunction has been linked to cerebral damage observed in subjects in critical condition, receiving high energy intake by PN^40^. Mitochondria are considered the cell’s powerhouse, because of their role in adenosine triphosphate (ATP) production through the oxidative phosphorylation of macronutrients. Mitochondrial function seems to be impaired in the critical illness, leading to reduction of biogenesis, increased reactive oxygen species generation, and decreased ATP synthesis up to 50%^40^. Despite interesting, these studies enrolled only adults and children in critical ill condition. Thus, no conclusions can be drawn with regard to preterm neonates and further studies are advocated to confirm this hypothesis in preterm newborns. This metabolic condition may contribute to brain damage. High energy intake in this phase may represent an inappropriate amount of energy for mitochondria with consequent increasing production of oxygen reactive, that may in turn worse brain impairment^40–42^. Further studies are advocated to confirm this crucial hypothesis in preterm newborns.

The results of this study should be interpreted taking into account specific limitations. The cUS is a highly operator-dependent imaging modality. In order to reduce bias, two different physicians performed a series of scans, and each measurement was recorded only after an agreement between them, unaware of the nutritional intake received at the time of the cUS examination. Inter- and intra-observer variability was not quantifiable in our study and this represent a limit of the study. The two ultra-sonographers measured each cerebral structure 3 times and, after agreeing on the adequacy of the measurement, reported in the data form only the mean value. Despite MRI is the gold-standard technique to measure cerebral structures, we used cUS for the evaluation of brain volumes. However, we collected and analyzed only measurements of the cerebral structures that were previously assessed in a comparative study between MRI and cUS, in order to improve the accuracy^43^. The use of cUS allowed us to perform serial measurements of brain structures, avoiding issues of transporting to the radiological service and the sedation during the scan session for this critical population. Moreover, it is not possible to establish if the results observed at 28 DOL on brain measurements may have consequences on NDV later in life. However, we have recently reported, in a similar population, that high energy intake in PN has been associated to a reduced NDV^5^. Finally, the low morbidity rate observed in our population, may limit the generatability of our results.

## Conclusion

Administering high doses of energy supply through PN may be harmful in premature neonates.

We suggest more wary approach in the administration of high energy intake by PN in preterm newborns during the first DOL. On the other hand, our results encourage the implementation of EN protocol as soon as possible. Further studies are warranted to establish the optimal energy intake to promote growth without adding adverse effects on the brain.

## Supplementary Information

Supplementary Figure S1.

Supplementary Figure S2.

Supplementary Figure S3.

Supplementary Tables.

## Data Availability

Relevant summary level statistics are presented in the manuscript. Individual-level data cannot be shared publicly because of privacy laws (Italian Law: D.Lgs. n. 196/2003). Data are available from Department of Maternal and Child Health Policlinico Umberto I, Sapienza University of Rome, Italy Institutional Data Access for researchers who meet the criteria for access to confidential data.

## Abbreviations

ATP: Adenosine triphosphate
BPD: Bronchopulmonary dysplasia
BW: Birth weight
cUS: Cranial ultrasonography
DOL: Days of life
EN: Enteral nutrition
GA: Gestational age
IVH: Intraventricular haemorrhage
MM: Maternal milk
MRI: Magnetic resonance imaging
NDV: Neurodevelopment
NEC: Necrotizing enterocolitis
NICU: Neonatal intensive care unit
PLV: Periventricular leukomalacia
PN: Parenteral nutrition
ROP: Retinopathy of prematurity
