# Supplementary material for: Effects of early energy intake on neonatal cerebral growth of preterm newborn: an observational study
Source: Sci Rep. 2021 Sep 16;11:18457. doi: 10.1038/s41598-021-98088-4 (PMC8445990; doi:10.1038/s41598-021-98088-4)
Supplement: Supplementary file 1 — Supplementary Figure S1. [file 41598_2021_98088_MOESM1_ESM.pdf]

Supplementary Figure 1. Cerebral measurements on cranial ultrasounds: Corpus Callosum.

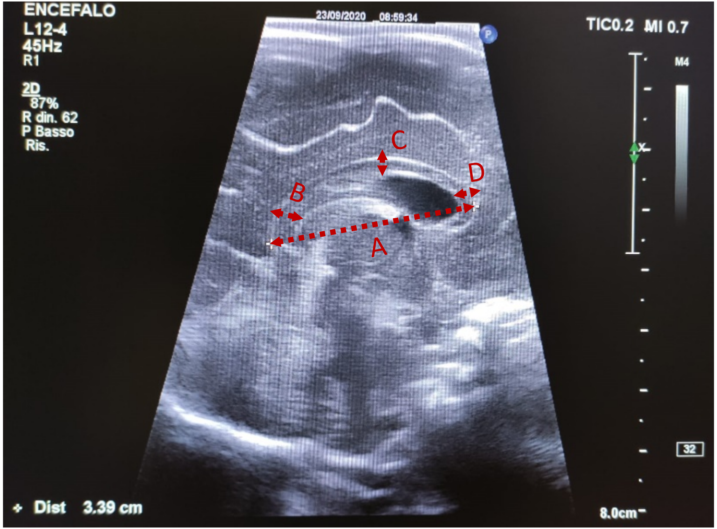

Figure Legend. Measurements of corpus callosum: (A) length; (B) splenium; (C) body; (D) genu.
