# Supplementary figures and images for: Effects of early energy intake on neonatal cerebral growth of preterm newborn: an observational study
Source: Sci Rep. 2021 Sep 16;11:18457. doi: 10.1038/s41598-021-98088-4 (PMC8445990; doi:10.1038/s41598-021-98088-4)

# Supplementary Figure 2. Cerebral measurements on cranial ultrasounds: Caudate Head Width.

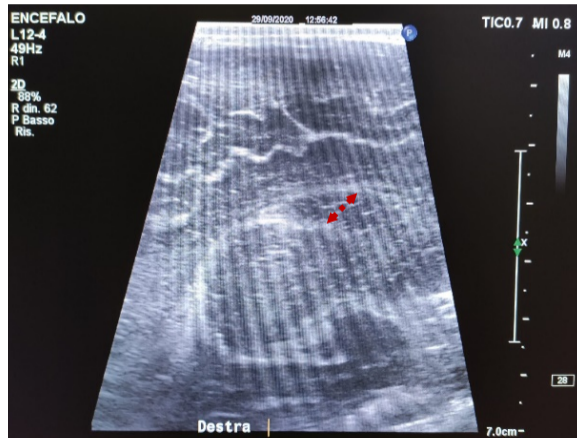

Supplement: Supplementary file 2 — Supplementary Figure S2. [file 41598_2021_98088_MOESM2_ESM.pdf]
