# Supplementary material for: Effects of early energy intake on neonatal cerebral growth of preterm newborn: an observational study
Source: Sci Rep. 2021 Sep 16;11:18457. doi: 10.1038/s41598-021-98088-4 (PMC8445990; doi:10.1038/s41598-021-98088-4)
Supplement: Supplementary file 3 — Supplementary Figure S3. [file 41598_2021_98088_MOESM3_ESM.pdf]

Supplementary Figure 3. Cerebral measurements on cranial ultrasounds: Cerebellum.

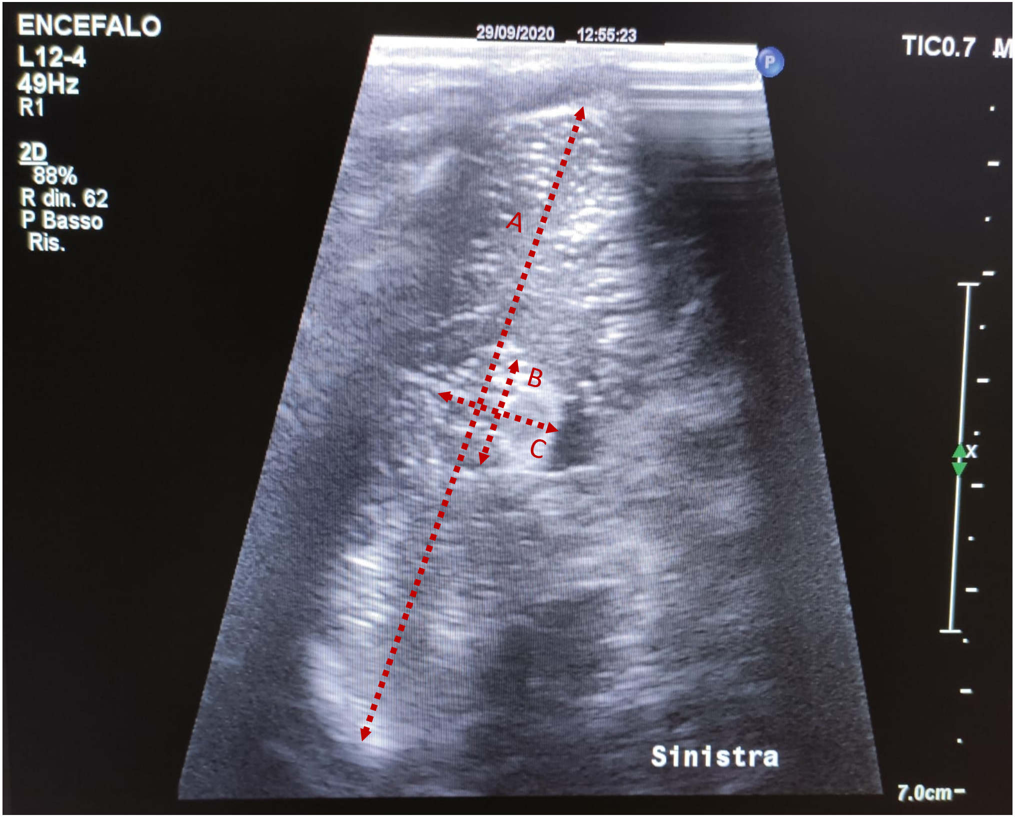

Figure Legend. Measurements of cerebellum: (A) Transverse diameter ; (B) Vermis Width ; (C) Vermis Height .
