# Supplementary material for: Effects of early energy intake on neonatal cerebral growth of preterm newborn: an observational study
Source: Sci Rep. 2021 Sep 16;11:18457. doi: 10.1038/s41598-021-98088-4 (PMC8445990; doi:10.1038/s41598-021-98088-4)
Supplement: Supplementary file 4 — Supplementary Tables. [file 41598_2021_98088_MOESM4_ESM.docx]

**Supplementary Table 1**. Parenteral Nutrition protocol.

|  |  | Birth weight  < 1000 g | Birth weight  ≥ 1000 g |
| --- | --- | --- | --- |
| Energy *(kcal/kg/day)* | Starting dose | 45 | 45 |
|  | Target dose | 105 | 100 |
| Proteins  *(g/kg/day)* | Starting dose | 2.0 | 2.0 |
|  | Target dose | 4.0 | 3.5 |
| Dextrose  *(g/kg/day)* | Starting dose | 7.0 | 7.0 |
|  | Target dose | 14.0 | 14.5 |
| Lipids  *(g/kg/day)* | Starting dose | 1.0 | 1.0 |
|  | Target dose | 3.5 | 3.0 |

**Notes.** Starting dose was administered at the age of 0 days of life, Target dose was reached at 7 days of life.

**Supplementary Table 2**. Cerebral size at birth of the study population.

| *Measures* | *N. 109* |
| --- | --- |
| **Corpus Callosum** |  |
| *Length* | 36.4 ± 3.8 |
| *Body* | 0.2 ± 0.1 |
| *Genu* | 0.3 ± 0.1 |
| *Splenium* | 0.3 ± 0.1 |
| **Caudate head** |  |
| *Right* | 0.5 ± 0.1 |
| *Left* | 0.5 ± 0.1 |
| **Cerebellum** |  |
| *Transverse diameter* | 3.7 ± 0.6 |
| *Vermis Height* | 0.9 ± 0.2 |
| *Vermis Width* | 0.6 ± 0.1 |

**Table legend:** Data were expressed as mean ± standard deviation.
